# Injury, intense dust exposure, and chronic disease among survivors of the World Trade Center terrorist attacks of September 11, 2001

**DOI:** 10.1186/s40621-017-0115-x

**Published:** 2017-07-17

**Authors:** Howard E. Alper, Shengchao Yu, Steven D. Stellman, Robert M. Brackbill

**Affiliations:** 1 New York City Department of Health and Mental Hygiene, 125 Worth Street, New York, 10013 USA; 2 Department of Epidemiology, Mailman School of Public Health, Columbia University, 722 W 168th St, New York, NY 10032 USA

**Keywords:** World trade center, 9/11, Disaster, Injury, Dust cloud, Chronic disease

## Abstract

**Background:**

The World Trade Center attack of September 11, 2001 in New York City (9/11) exposed thousands of people to intense concentrations of hazardous materials that have resulted in reports of increased levels of asthma, heart disease, diabetes, and other chronic diseases along with psychological illnesses such as post-traumatic stress disorder (PTSD). Few studies have discriminated between health consequences of immediate (short-term or acute) intense exposures versus chronic residential or workplace exposures.

**Methods:**

We used proportional hazards methods to determine adjusted hazard ratios (AHRs) for associations between several components of acute exposures (e.g., injury, immersion in the dust cloud) and four chronic disease outcomes: asthma, other non-neoplastic lung diseases, cardiovascular disease, and diabetes, in 8701 persons free of those conditions prior to exposure and who were physically present during or immediately after the World Trade Center attacks. Participants were followed prospectively up to 11 years post-9/11.

**Results:**

Heart disease exhibited a dose-response association with sustaining injury (1 injury type: AHR =2.0, 95% CI (Confidence Interval) 1.1–3.6; 2 injury types: AHR = 3.1, 95% CI 1.2–7.9; 3 or more injury types: AHR = 6.8, 95% CI 2.0–22.6), while asthma and other lung diseases were both significantly associated with dust cloud exposure (AHR = 1.3, 95% CI 1.0–1.6). Diabetes was not associated with any of the predictors assessed in this study.

**Conclusion:**

In this study we demonstrated that the acute exposures of injury and dust cloud that were sustained on 9/11/2001 had significant associations with later heart and respiratory diseases. Continued monitoring of 9/11 exposed persons’ health by medical providers is warranted for the foreseeable future.

## Background

The terrorist attacks on the World Trade Center (WTC) in New York City on September 11, 2001 (9/11) left nearly 3000 people dead and many thousands injured. The collapse of the towers created a massive dust and debris cloud which enveloped many survivors and ultimately led to the development of a variety of physical and mental diseases (Brackbill et al. 2006; Brackbill et al. 2009; Farfel et al. 2008; Perlman et al. 2011). The most heavily exposed groups included occupants of buildings, passersby, residents of lower Manhattan, and early responding rescue/recovery workers. These groups were exposed to numerous toxic components of the dust and debris cloud including fine particulate matter, caustic dusts, volatile organic compounds, and heavy metals, as well as combustion products of the fires that followed the initial impact (Landrigan et al. 2004). Many individuals also sustained injuries, as well as traumatic experiences such as witnessing planes hit the building, people falling or jumping from buildings, and seeing people injured or killed (Brackbill et al. 2006).

Multiple studies have found elevated rates of asthma, other lung conditions, and post-traumatic stress disorder (PTSD) among rescue/recovery workers, especially early responders, residents of lower Manhattan, and other people who were in the vicinity, persisting to at least 6 years post-disaster (Banauch et al. 2006; Brackbill et al. 2006; Brackbill et al. 2009; Lin et al. 2007). Physical health outcomes that have been linked to exposures on the morning of 9/11 include asthma and other respiratory illnesses (Brackbill et al. 2009), heart disease (Jordan et al. 2011), and potentially diabetes (Miller-Archie et al. 2014). Numerous studies have found 9/11 trauma to be associated with subsequent development of PTSD, often comorbid with respiratory disease (Friedman et al. 2013), with the risk for 9/11 related asthma and heart disease greater in those with PTSD (Brackbill et al. 2014; Jordan et al. 2011).

In this study we focus on physical trauma that occurred on the morning of 9/11/2001 during or as an immediate result of the attacks, often in association with the extreme environmental pollution engendered by the dust/debris cloud created during the collapse of the towers and other buildings. The maximum concentration of particulate matter within the dust/debris cloud on 9/11 has been estimated to be 100,000 μm per cubic meter of particles (Lioy and Georgopoulos 2006). Potential for such acute exposure to individuals lasted about 5 to 6 hours after the plane attacks as the cloud was quickly advected into the lower atmosphere by the heat of the fires and moved southeasterly over Brooklyn, eventually dispersing over the ocean. It is estimated that 90% of the total amount of environmental material resulting from fires and three building collapses (WTC 1, 2, and 7) was released and present on 9/11. Indoor chronic exposures occurring in the months following 9/11 have also not been well quantified, but contamination of office buildings and residences by dust and fire products have been qualitatively reported as substantial (Lioy and Georgopoulos 2006). One group of individuals who directly experienced acute as distinct from chronic exposures on 9/11 were people in the vicinity of the buildings at the time of the attacks either as passersby or occupants of the towers and surrounding buildings. It is to be noted that shortly after the collapse of the buildings the entire area of Lower Manhattan up to 14^th^ Street was ordered evacuated except for rescue and recovery personnel; this so-called restricted zone encompassed most of downtown Manhattan until the end of September, 2001, limiting the number of persons potentially exposed to either outdoor or indoor chronic exposure for a month after the event.

Few studies have examined single exposure-disease associations (e.g., dust cloud and asthma, injury and heart disease) limited to persons who were primarily exposed on 9/11/2001 (Brackbill et al. 2006; Brackbill et al. 2014). For instance, Brackbill (2006) examined the prevalence of respiratory symptoms among persons who had been an evacuee of a collapsed building and caught in resultant dust debris cloud, and they also (2014) examined associations between sustaining an injury and/or being enveloped in the intense dust cloud with heart and respiratory disease among persons who were south of Chambers Street on 9/11/2001. The present study extends knowledge of the effects of the 9/11 attacks by investigating the association between acute exposure to 9/11 and chronic disease 10–11 years post event, employing methods appropriate to the longitudinal nature of the data.

In the present study, we estimate the relative hazard of reporting diagnosed angina or heart attack, diabetes, asthma or other lung conditions in the WTC Health Registry cohort up to 11 years after the event. We focused on Registry enrollees who experienced acute, direct exposure to dust/debris cloud from the collapse of buildings or traumatic injury on the morning of 9/11/2001. We did not include in the study sample residents who returned to live in downtown Manhattan after 9/11 or rescue/recovery workers who worked at the site after 9/11. We hypothesized that for this narrowly defined cohort, injury would be associated with heart conditions, and dust/debris cloud with respiratory conditions, with no expected association of acute 9/11 exposures with diabetes.

## Methods

The WTC Health Registry monitors the physical and mental health of 71,431 persons exposed to the WTC attacks on September 11, 2001 (Farfel et al. 2008). Enrollees were recruited from lists of potentially exposed persons provided by employers and businesses (list-identified), as well as from media outreach and a toll-free number (self-identified). Rescue/recovery workers, residents of lower Manhattan, area workers, passersby on the streets or subway, and students or staff of neighborhood schools were eligible for inclusion. These groups were defined so as to be mutually exclusive. The Registry conducted health surveys among enrollees in 2003–2004 (wave 1), 2006–2007 (wave 2), and 2011–2012 (wave 3). For wave 1, most participants (95%) were interviewed using computer-assisted telephone interview, and the rest were interviewed in person (5%). For waves 2 and 3, enrollees self-administered their interviews via internet or mail or were interviewed by phone. The methods of the Registry have been described in detail in previous publications (Brackbill et al. 2009; Farfel et al. 2008). The Registry was approved by the institutional review boards of the Centers for Disease Control and Prevention and the New York City Department of Health and Mental Hygiene.

### Study sample

People south of Chambers Street in lower Manhattan on September 11, 2001 were most likely to have been injured or immersed in the dust cloud from the collapse of the buildings. We defined acute exposures as being present near the disaster site (i.e., south of Chambers Street) on 9/11, but not afterwards, to differentiate acute from chronic exposures. We limited our study sample to enrollees who had completed all three Registry survey waves in order to maximize follow-up time and to capture relevant exposure variables. Our definition of acute exposure resulted in different exclusion rules for the four eligibility groups. Rescue/recovery workers who worked at the WTC site after the first day of the disaster (*n* = 2917) and residents who did not evacuate or evacuated but returned to their homes before December 31, 2001 (*n* = 1508) were excluded. For area workers whose normal workplaces were in the defined acute exposure zone and who were at work on 9/11, we retained those who indicated they returned to work after 9/11, so that we could compare the association of acute exposures on 9/11 with health outcomes for a similar population who may have had more chronic exposures from returning to the area. There was no exclusion for passersby on 9/11 because presumably this group was exposed to 9/11 on the disaster day only.

Adults older than 64 years on 9/11 (*n* = 244) were excluded because it would be considerably more difficult to distinguish effects of 9/11 exposure on chronic disease from those associated with age. Finally, to focus on post-9/11 incidence of chronic diseases, we excluded persons who reported having been diagnosed by a health professional with any of the following physical health conditions before 2002: heart attack, angina, other heart conditions, asthma, chronic bronchitis, emphysema, reactive airway disease, other lung conditions, stroke, diabetes, sarcoidosis, or cancer (*n* = 10,796). The resulting study sample included 8701 individuals, consisting of 7503 area workers; 249 rescue/recovery workers; 131 residents; and 818 passersby.

### Study variables

Socio-demographic characteristics of study sample, such as gender, age (age on 9/11/2001), race/ethnicity, and education, were included in the analytical models. Study recruitment source, indicating whether Registry enrollees were self-enrolled or recruited through lists provided by employers or businesses, was also controlled as a potential source of selection bias. Risk factors for chronic diseases included hypertension, smoking, probable PTSD at Wave 1, and body mass index (BMI). Hypertension was defined as answering “yes” to the question “Have you ever been told by a doctor or other health professional that you had hypertension or high blood pressure?” at either wave 1 or wave 2. Smoking status was classified as “current”, “former”, or “never”, and was derived from “ever smoke” and “smoke now” questions from the wave 1 and wave 2 surveys. BMI was calculated from height and weight, which were reported by enrollees at wave 3 only. BMI was categorized as underweight (<18.5 kg/m^2^), normal weight (18.5- < 25 kg/m^2^), overweight (25- < 30 kg/m^2^), and obese (≥30 kg/m^2^), as per Centers for Disease Control and Prevention guidelines. Since a small fraction of the study sample was underweight (<1%), there would be insufficient statistical power to investigate this stratum, so these enrollees were excluded from analysis.

Acute 9/11 exposure in this study was measured by two variables: number of injury types and dust cloud exposure. Similar to an earlier Registry study (Brackbill et al. 2014), number of types of injuries was defined by whether respondents reported at wave 1 sustaining any of the following types of injury on 9/11: cut, abrasion or puncture wound, sprain or strain, burn, broken bone or dislocation, and concussion or head injury. The part of the body affected was not recorded; therefore the number of injury types is simply the number of types the respondent reported, classified as 0, 1, 2, or 3 or more injury types.

Acute dust cloud exposure on 9/11 was based on questions from the wave 1 and wave 2 surveys and was classified as intense versus some or none. Intense exposure was defined as having been in the dust cloud on 9/11 and reporting at least one of five experiences: being unable to see more than a few feet; having difficulty walking or finding one’s way; trouble finding shelter; being covered with dust; or not being able to hear. The “some or none” category consisted of those who had reported being in the dust/debris cloud at wave 1 but who did not experience intense exposure or reported no dust cloud exposure at all. We addressed discrepancies between wave 1 and wave 2 on dust cloud reporting by categorizing persons who said they were not in dust cloud on wave 1 as “none”, regardless of how they answered dust cloud questions on wave 2. If they were affirmative for dust cloud or missing on wave 1, we applied the information they reported on wave 2. Because arriving on 9/11 as a rescue/recovery worker is an established risk factor for a number of health outcomes, particularly lung problems (Brackbill et al. 2009; Prezant et al. 2002), we included being a rescue/recovery vs. not being a rescue/recovery worker variables in the models to account for previously reported increased risk for this group.

Probable PTSD was assessed at wave 1 using the PTSD Checklist-Specific (PCL-S), a 17-item self-reported symptom scale which referred specifically to the events of September 11. The 17 items correspond to Diagnostic and Statistical Manual of Mental Disorders (DSM-IV) PTSD symptoms [DSM-IV 1994]. In this scale enrollees rate the degree to which they were bothered by symptoms in the past 30 days (1 = not at all, to 5 = extremely). Responses to the 17 items are summed, giving a possible total score of 17 to 85. Probable PTSD (herein referred to simply as PTSD) was defined as a PCL score ≥ 44. The PCL-S is a well-validated measure and has good temporal stability, internal consistency (>0.75), test-retest reliability (0.66), and high convergent validity (0.58–0.93) (Wilkins et al. 2011), with overall diagnostic efficiency = 0.90, sensitivity = 0.94, and specificity = 0.86 (Blanchard et al. 1996).

Four distinct chronic disease endpoints were studied: angina/heart attack, asthma, non-neoplastic lung diseases other than asthma, and diabetes as reported at waves 2 or 3. All disease endpoints were by self-report. The wave 2 and wave 3 follow-up questionnaires each contained a checklist of medical conditions headed by the question “Have you ever been told by a doctor or other health professional that you had any of these conditions?” Respondents also reported year of diagnosis. The diagnosis year of a heart condition at either wave was taken as the earlier of the years reported for angina and heart attack. Due to differences in questionnaire wording non-neoplastic lung disease was defined at wave 2 by self-report of chronic bronchitis, emphysema, or “other lung conditions,” and at wave 3 by self-report of chronic bronchitis, emphysema, or pulmonary fibrosis. The time of onset for non-neoplastic lung disease at either wave was defined to be the earliest date reported for any of the lung diseases.

### Statistical analysis

For each chronic disease endpoint, the time to event and censoring status were determined by the following algorithm. Person time began on the date of enrollment (2003–2004) into the Registry. If the enrollee reported a single chronic disease, this was considered the endpoint, and the event was treated as uncensored. If the enrollee experienced multiple chronic diseases, the first was considered the endpoint. Cancer incidence has been reported twice previously in this cohort (Li et al. 2012; Li et al. 2016) and was therefore treated as a censoring event but not an endpoint of interest; i.e. if cancer occurred before any other disease, the year of diagnosis was the censoring date. Occurrence of cancer was determined via linkage to the cancer registries of New York, New Jersey, Connecticut, and eight other states that together account for at least 95% of all new cancer cases in the Registry (Li et al. 2012). If the enrollee did not experience any of the foregoing chronic diseases, follow-up was terminated at the date of completion of the wave 3 survey. Enrollees reported only the year of chronic disease diagnosis; therefore, the date of chronic disease diagnosis was set to June 30^th^ of the year of disease report, if different from year of enrollment in the Registry. If year of disease diagnosis occurred in the year of enrollment, the date was set to December 31^st^ of that year.

Cox proportional hazards regression methods were employed to investigate the association between number of injury types, dust/debris cloud exposure, being a rescue/recovery worker, and having probable PTSD at wave 1, with chronic disease, adjusting for potential confounders that included gender, age, race/ethnicity, education, BMI, and study recruitment source (list vs. self-identified). PTSD at wave 1 was included as a risk factor in the multivariable model because it has been shown to be a contributing risk factor to the outcomes of interest in this study including heart disease (Jordan et al. 2011) and lung problems (Friedman et al. 2016). Multivariable analyses also included smoking status (current/former/never), and ever reporting hypertension. Separate regressions were performed for each of the four endpoints: angina/heart attack, asthma, other non-neoplastic lung diseases, and diabetes. Possible dose-response relationships were studied using number of injury types as a predictor variable.

We conducted a separate regression analysis among area workers who did and who did not return to their lower Manhattan workplaces between September 12 and December 31, 2001. A similar regression analysis was also run for passersby alone. Due to small numbers, rescue/recovery workers and residents were not analyzed separately.

The proportional hazards assumption was tested for the number of injury types, dust cloud exposure, and rescue/recovery worker status by creating interaction terms between each of these variables and log (time to event), and testing the statistical significance of these interactions, separately for each chronic disease endpoint.

Statistical tests were considered significant if the associated p-value was less than 0.05. All analyses were performed using SAS 9.4 (SAS Institute, Inc., Cary, North Carolina).

## Results

A total of 8701 enrollees met the inclusion criteria; they comprise 12.8% of all adult Registry enrollees. Characteristics of the study sample are shown in Table 1. Participants were mostly male (51%), aged 25–44 at 9/11 (56%), non-Hispanic white (69%), and college-educated or more (67%). The majority (82%) were recruited into the Registry via self- rather than list-enrollment. BMI was distributed as normal weight (32%), overweight (40%), and obese (28%). One-tenth of the participants were current smokers (10%), while 26% reported a history of high blood pressure; 41% had intense exposure to the dust cloud. The majority of the sample (87%) experienced no injuries, while 10% had a single injury, 2% had two injuries, and 1% experienced 3 or more injuries on 9/11. Symptoms indicative of probable PTSD at wave 1 were reported by 14% of the sample. The majority of the study population was area workers (86%); another 9% were passersby. A total of 92 incident cases of heart disease were reported during the follow-up period, as well as 327 new cases of diabetes, 308 of asthma, and 297 of other non-neoplastic lung diseases.

**Table 1:** Demographic, Behavioral, and Exposure Characteristics of WTCHR enrollees who were South of Chambers Street on 9/11/2001, World Trade Center Health Registry, 2003-2012^a^

Characteristics	All	
	N	%
Total	8701	100.0
Gender		
Male	4457	51.2
Female	4244	48.8
Age at 9/11, Years		
18–24	472	5.4
25–44	4867	56.0
45–54	2537	29.2
55+	814	9.4
Race/ethnic group		
Non-Hispanic White	6008	69.1
on-Hispanic Black	1117	12.8
Hispanic	861	9.9
Asian	475	5.5
Other	240	2.8
Education		
Less than high school or high school	1172	13.5
Some college	1661	19.2
College or post grad	5830	67.3
Study Recruitment Source		
List	1595	18.3
Self	7106	81.7
Body Mass Index		
Normal Weight	2690	31.8
Overweight	3356	39.7
Obese	2410	28.5
Smoking		
Never	5019	58.8
Former	2710	31.7
Current	813	9.5
High Blood Pressure		
Yes	2300	26.4
No	6401	73.6
Dust Cloud Exposure		
Intense	3595	41.3
Some/None	5106	58.7
Number of Injury Types		
None	7604	87.4
1	854	9.8
2	185	2.1
3+	58	0.7
PTSD at Wave 1		
Yes	1197	14.0
No	7342	86.0
Eligibility Group		
Rescue/Recovery Worker	249	2.9
Resident	131	1.5
Area Worker	7503	86.2
Passersby	818	9.4
Student/Staff	0	0.0

^a^Number of missing values: Gender 0, Age 11, Race/Ethnicity 0, Education 38, Recruitment Source 0, Rescue/Recovery Worker 0, Resident 0, BMI 173, Smoking 159, High Blood Pressure 0, Dust Cloud 0, Number of Injuries 0, PTSD 162

The results of the Cox Proportional Hazards regressions are presented in Tables 2 and 3. The proportional hazards assumption was satisfied for all the exposures used in the models.

**Table 2:** Predictors of diagnosed chronic disease for directly exposed WTCHR enrollees (*n* = 8701), World Trade Center Health Registry, 2003–2012

Characteristic	Angina/Heart Attack^a^	Diabetes^a^	Asthma^a^	Other Non-Neoplastic Lung Diseases^a,b^
	AHR (95% CI)	AHR (95% CI)	AHR (95% CI)	AHR (95% CI)
Number of Events	92	327	308	297
Number of Injury Types				
0	Ref.	Ref.	Ref.	Ref.
1	2.0 (1.1, 3.6)	0.9 (0.7, 1.4)	1.3 (0.9, 1.8)	0.7 (0.5, 1.0)
2	3.1 (1.2, 7.9)	0.8 (0.4, 1.7)	0.8 (0.4, 1.9)	0.7 (0.4, 1.5)
3+	6.8 (2.0, 22.6)	0.8 (0.2, 3.0)	0.8 (0.2, 3.4)	0.3 (0.0, 2.4)
Dust Cloud Exposure				
Intense	0.8 (0.5, 1.3)	1.1 (0.9, 1.4)	1.3 (1.0, 1.6)	1.3 (1.0, 1.6)
Some/None	Ref	Ref	Ref	Ref
PTSD at Wave 1				
Yes	0.9 (0.5, 1.6)	0.9 (0.7, 1.3)	1.1 (0.8, 1.5)	1.6 (1.2, 2.1)
No	Ref	Ref	Ref	Ref
Rescue/Recovery Worker				
Yes	0.7 (0.2, 2.4)	0.9 (0.4, 1.7)	1.7 (0.9, 3.0)	1.8 (1.0, 3.1)
No	Ref	Ref	Ref	Ref
Body Mass Index				
Normal Weight	Ref	Ref	Ref	Ref
Overweight	1.3 (0.8, 2.3)	1.7 (1.2, 2.4)	1.1 (0.8, 1.5)	0.9 (0.7, 1.3)
Obese	1.3 (0.7, 2.3)	3.5 (2.5, 4.9)	1.5 (1.1, 2.0)	1.3 (0.9, 1.7)
Smoking				
Current	1.5 (0.7, 3.0)	1.3 (0.9, 1.9)	0.8 (0.6, 1.3)	2.1 (1.5, 2.9)
Former	1.6 (1.0, 2.6)	1.1 (0.9, 1.4)	1.2 (0.9, 1.5)	1.1 (0.9, 1.5)
Never	Ref	Ref	Ref	Ref
High BP at Wave 1 or 2				
Yes	2.0 (1.3, 3.2)	2.6 (2.0, 3.2)	1.1 (0.9, 1.5)	1.3 (0.9, 1.6)
No	Ref	Ref	Ref	Ref

*Abbreviations*: *AHR* adjusted hazard ratio, *CI* confidence interval, *PTSD* posttraumatic stress disorder, *BP* blood pressure, *BMI* Body Mass Index

^a^Multivariable model is adjusted for all variables shown plus study recruitment source, age, gender, race/ethnicity, and education

^b^Other Non-Neoplastic Lung Diseases include chronic bronchitis, emphysema, pulmonary fibrosis, and other lung conditions

**Table 3:** Predictors of diagnosed angina/heart attack and asthma for those directly exposed ^a^, including area workers stratified by whether they returned or did not return to work, and passersby^a^, South of Chambers Street before December 31, 2001, World Trade Center Health Registry, 2003–2012

	Area Workers				Passersby	
	Returned to Work (*N* = 5318)		Did Not Return to Work (*N* = 2185)		(*N* = 818)	
Characteristics	Angina/Heart Attack^b^	Asthma^b^	Angina/Heart Attack^b^	Asthma^b^	Angina/Heart Attack^b^	Asthma^b^
	AHR (95% CI)	AHR (95% CI)	AHR (95% CI)	AHR (95% CI)	AHR (95% CI)	AHR (95% CI)
Number of Events	57	190	21	66	11	26
Number of Injury Types						
0	Ref.	Ref.	Ref.	Ref.	Ref.	Ref.
1	2.1 (0.9, 4.8)	1.7 (1.1, 2.6)	1.2 (0.3, 4.4)	0.5 (0.2, 1.1)	8.1 (2.0, 33.0)	1.8 (0.5, 6.2)
2	2.8 (0.7, 11.9)	0.6 (0.1, 2.3)	2.7 (0.5, 13.2)	1.4 (0.5, 4.0)	0.0	0.0
3+	18.3 (4.1, 82.2)	2.9 (0.7, 11.8)	9.0 (0.98, 83.5)	0.0	0.0	0.0
Dust Cloud Exposure						
Intense	0.8 (0.4, 1.3)	1.1 (0.8, 1.5)	1.1 (0.4, 2.9)	2.0 (1.2, 3.3)	0.9 (0.2, 3.3)	0.8 (0.3, 1.9)
Some/None	Ref	Ref	Ref	Ref	Ref.	Ref.
PTSD at Wave 1						
Yes	0.9 (0.4, 2.0)	1.1 (0.8, 1.7)	0.9 (0.3, 3.2)	0.8 (0.4, 1.6)	1.3 (0.3, 6.4)	0.9 (0.3, 3.1)
No	Ref	Ref	Ref	Ref	Ref.	Ref.

^a^Rescue/Recovery Workers and residents not included because of small sample size

^b^Multivariable model is adjusted for all variables shown plus BMI, smoking, high blood pressure, study recruitment source, age, gender, race/ethnicity, and education

### Angina/heart attack

Angina/Heart Attack was significantly associated with the number of injury types: for 1 injury the adjusted hazard ratio (AHR) = 2.0, 95% confidence interval (CI) 1.1–3.6; for 2 injuries, AHR = 3.1, 95% CI 1.2–7.9; for 3 or more injuries AHR = 6.8, 95% CI 2.0–22.6. The association demonstrated a significant dose-response relationship (*p* < 0.0001). Current cigarette smoking was not a significant predictor, but the prevalence of current smoking in the study population is less than 10% so the number of new cases among smokers was very small. Being a former smoker was significantly associated with angina/heart attack (AHR = 1.6, 95% CI 1.0–2.6). Angina/heart attack was also significantly related to high blood pressure at either wave 1 or wave 2 (AHR = 2.0, 95% CI 1.3–3.2).

### Diabetes

Diabetes did not exhibit a statistically significant association with either injury or dust cloud exposure, nor with PTSD at wave 1. Diabetes had a statistically significant dose-response relationship with BMI (overweight: AHR = 1.7, 95% CI 1.2–2.4; obese: AHR = 3.5, 95% CI 2.5–4.9) (dose response *p* < 0.0001). Diabetes was significantly related to high blood pressure reported at either wave 1 or wave 2 (AHR = 2.6, 95% CI 2.0–3.2).

### Asthma

Asthma was related to dust cloud exposure (AHR = 1.3, 95% CI 1.0–1.6), and demonstrated a dose-response relationship with BMI (overweight: AHR = 1.1, 95% CI 0.8–1.5, obese: AHR = 1.5, 95% CI 1.1–2.0) that was not statistically significant.

### Non-neoplastic lung diseases

Non-neoplastic lung diseases were significantly associated with dust cloud exposure (AHR = 1.3, 95% CI 1.0–1.6), but not with injury. Non-Neoplastic lung diseases were also associated with PTSD (AHR = 1.5, 95% CI 1.1–2.0), and with rescue/recovery worker status (AHR = 1.8, 95% CI 1.0–3.1), and current smoking (AHR = 2.1, 95% CI 1.5–2.9).

### Area workers and passersby

Sufficient numbers of area workers were available to permit comparison of exposure-outcome relationships between those who eventually returned to their workplaces with those who did not. Results are shown in Table 3. For area workers who returned to a workplace south of Chambers Street between September 12 and December 31, 2001, angina/heart attack was significantly associated with 3 or more injury types (AHR = 18.3, 95% CI 4.1–82.2), and the overall injury-angina/heart attack association exhibited a dose response relationship (*p* = 0.0003). Asthma exhibited a significant association with 1 injury type (AHR = 1.7, 95% CI 1.1–2.6).

For area workers who did not return to work south of Chambers Street, angina/heart attack was associated with 3 or more injuries, but not significantly (possibly due to small number of diagnoses) (AHR = 9.0, 95% CI 0.98–83.5), while the overall injury-angina/heart attack association did not exhibit a dose response relationship (*p* = 0.066). Asthma was significantly associated with dust cloud exposure (AHR = 2.0, 95% CI 1.2–3.3).

Table 3 also shows exposure disease relationships for passersby. Among this group angina/heart attack was significantly associated with 1 injury type only (AHR = 8.1, 95% CI 2.0–33.0) while the risk of asthma for those with 1 injury type was nearly two-fold, although not significant (AHR = 1.8, 95% CI 0.5–6.2); however the total number of asthma diagnoses was small (*n* = 26).

## Discussion

The present study is a unique opportunity to investigate the long-term health consequences of a very short-term exposure, an opportunity that is not typically available after events such as 9/11. We observed increased risk of several chronic diseases in persons with predominantly acute exposure to specific hazards related to the 9/11 attacks. In particular, 9/11 related injuries were strongly predictive of angina/heart attack, while intense environmental dust cloud exposure on 9/11 and 9/11-related PTSD were predictive of non-neoplastic lung diseases. In this group of initially relatively healthy persons who were south of Chambers Street on 9/11/2001, we found a significant dose-response association between number of injury types and angina/heart disease, but not with asthma, other non-neoplastic lung diseases, or diabetes. Dust/debris cloud exposure was associated with asthma and other non-neoplastic lung diseases but not angina/heart disease. Using generally accepted estimates of the potentially exposed population, our data suggest that approximately 51,000 people (of 300,000 people below Chambers St. on 9/11/2001 (Murphy et al. 2007)) could develop a chronic disease up to 11 years after the 9/11 attacks.

The present analysis extends previous Registry findings regarding injury and heart disease. Using follow-up data through 2007 (i.e., six years after the attacks), Jordan (Jordan et al. 2011) found an increased risk of heart disease among adult enrollees, both male and female, for injury sustained on 9/11 (AHRs 1.33 and 1.46, respectively), while Brackbill et al. (Brackbill et al. 2014) showed a dose-response relationship (e.g., adjusted odds ratio = 2.2 with 1 injury and 3.3 with 2 injuries for heart disease [angina, heart attack, and other heart disease]) between number of types of injuries and heart disease for enrollees aged 18 and older who were south of Chambers St. on 9/11. In the present study, the magnitude of the risk estimate of heart attack or angina up to 11 years post-9/11 was greater with an AHR = 6.8 for 3 injuries sustained on 9/11/2001 for a subgroup of persons with primarily acute exposures on 9/11/2001. In addition, the independent association between injury and heart attack/angina in the absence of PTSD is similar to the report of a similar relationship with cardiovascular outcomes 5 to 6 years after 9/11 (Brackbill et al. 2014).

Nonetheless, traumatic injury is frequently a principal event leading to PTSD, but the mechanisms by which injury, PTSD, or both may be precursors of heart disease and other chronic illnesses require further elucidation. Biological mechanisms have been proposed to explain the relationship between PTSD and heart disease (Boscarino 2004; Kubzansky et al. 2007) involving primarily neuro-endocrinologic alterations leading to impaired adaptation to stress (Kubzansky and Koenen 2009). In addition, there are multiple other ways in which a traumatic injury can contribute to chronic illness later in life. Some may involve specific biological mechanisms, such as impact on organ systems (e.g., intracranial injury is often associated with hypertension), while the temporary or long-term loss of function due to musculoskeletal injuries may affect behaviors such as exercise and diet that are themselves risk factors for cancer, heart disease, and other chronic illnesses. In the Nurse’s Health Study, for example, it has been observed that traumatic events (including injury to one’s self) were significantly associated with cardiovascular disease (defined as either myocardial infarction or stroke) in the absence of PTSD symptoms (Sumner et al. 2015).

Our results pertain primarily to persons who were present on 9/11 during attacks and could have experienced a range of injury trauma and/or intense environmental pollution from the dust/debris cloud. Some of the area workers in the study sample likely experienced additional exposures after returning to contaminated workplaces that had not been thoroughly cleaned, but these additional exposures would have been small in comparison to those experienced on 9/11. For instance, among persons who returned to work after 9/11, 70% of those who had intense dust cloud exposure did not report heavy dust in their workplace (based on wave 2 question on damage to workplace). Such workers were obviously sufficiently healthy to return to work and likely had less respiratory conditions than those who did not return, which is one possible explanation for the differences in respiratory outcomes between the two groups.

Injuries that were sustained on 9/11 were significantly associated with heart disease for a sub group of area workers who returned to work after 9/11, but who were also limited to returning to areas some distance from the WTC site. Injuries also were associated with heart disease but not statistically significant for persons who did not return to the area. We believe that the difference in the results between the two sub groups of workers was due to a reduced sample for the non-returning worker group. In a separate sensitivity analysis, we added persons who had not returned for a month after 911 to the non-return group and found a significant association between injury and heart disease. Our results also suggest that the physical effects of having an injury did not necessarily prevent people from returning to work, but that injury nonetheless was associated with increased likelihood of heart attack or angina for persons who were physically present during the attacks on 9/11.

In this analysis, we used intense dust cloud that resulted from the two collapsing buildings in the morning of 9/11 as the dominant acute respiratory related exposure and we found it to be significantly associated with asthma and non-neoplastic lung diseases. These findings on acute pollution exposure on 9/11 are consistent with other analysis showing that persistent lower respiratory symptoms are strongly associated with a composite measure that included dust cloud exposure as well as experiencing or witnessing other traumatic events, which are ostensibly acute exposures (Friedman et al. 2016). Other studies have reported the association between clinically based measures of lung function with arriving on 9/11/2001 among rescue workers up to 6 years after 9/11/2001 (Aldrich et al. 2010; Wisnivesky et al. 2011) and between dust or odor present more than 3 months with lower respiratory symptoms 2 years after 9/11/2001 among residents in lower Manhattan (Lin et al. 2010; Reibman et al. 2005).

PTSD was significantly associated only with non-neoplastic lung diseases other than asthma. This finding is consistent with comorbidity of PTSD and lower respiratory symptoms that has been reported for both rescue/recovery workers (Friedman et al. 2013; Luft et al. 2012) and non- rescue/recovery workers (Nair et al. 2012). However, an association between PTSD and heart attacks/angina was not observed, in contrast to a previous Registry study (Jordan et al. 2011). A putative explanation is that by limiting the sample to persons who were healthy prior to 2002, we excluded unhealthy persons who exhibited a stronger PTSD-heart disease association. We performed a sensitivity analysis to address this hypothesis by returning to our analytic sample the ~11,000 enrollees with chronic disease before 2002. We found hazard ratios closer to the results of Jordan et al., though just shy of statistical significance. The lack of statistical significance is likely due to the fact that Jordan et al. included enrollees north of Chambers Street, whereas we did not.

Injury, dust cloud exposure, and rescue/recovery worker status were not associated with incident diabetes. The significant association between PTSD and diabetes previously reported by Miller-Archie et al. in the complete Registry cohort was not observed in this sample (Miller-Archie et al. 2014). Similar to the above discussion of PTSD-Heart Disease association, we hypothesized that this difference was likely due to the inclusion of enrollees with pre-9/11 histories of chronic diseases other than diabetes in the earlier study of Miller-Archie et al. We performed sensitivity analyses which demonstrated that reinstatement of enrollees with chronic disease before 2002 in our analytic sample produced results very similar to those of Miller-Archie et al. 2014.

### Strengths and limitations

A major strength of this study is that the large sample size allowed us to investigate the effect of acute exposure to 9/11 on a range of chronic diseases even while imposing extensive restrictions on dates of presence at the WTC site and prior medical history. The cohort has aged more than 11 years since the acute 9/11 exposures took place, providing an expectation of a natural increase in the frequency of chronic disease endpoints.

An important limitation is lack of information on injury severity, location in the body, whether the injury was treated in an emergency department or hospital, or the circumstances of the injury, leading us to use the number of types of injuries as a proxy for injury severity. However, there is evidence that more than one type of injury is associated with increased risk of death and longer stays in hospital (Aharonson-Daniel et al. 2003).

Data on both exposures and outcomes such as number of injuries, dust cloud exposure, PTSD symptoms, and chronic disease diagnosis, were based on self-report, which is subject to mis-reporting. However, there is substantial concordance between many Registry findings and clinical studies of World Trade Center survivors by other groups, as well as internal validation studies of cancer (Li et al. 2016). Furthermore, the association between PTSD and heart disease based on self-report (Jordan et al. 2011) is in good agreement with results based on hospital discharge records (Jordan et al. 2013).

A further limitation was loss to follow-up between surveys, since 68 and 63% of the original sample completed waves 2 and 3, respectively. Attrition may have affected the present results, but this effect was found to be negligible in a previous Registry study (Yu et al. 2015).

## Conclusion

The present study provides evidence that acute/direct exposure to the events of September 11, 2001 is associated with chronic disease 11 years after the disaster. More specifically, sustaining an injury was associated with subsequent heart disease, and exposure to the dust cloud was associated with respiratory disease. An implication of the present study is that significant numbers of people who were present on 9/11 could be vulnerable to developing chronic diseases many years later. Clinicians should be aware of the potential risk of chronic disease among those exposed to the World Trade Center attacks.

## Abbreviations

AHR: Adjusted hazard ratio
BMI: Body mass index
CI: Confidence interval
DSM-IV: Diagnostic statistical manual-IV
PCL-S: Posttraumatic stress disorder check list - specific
PTSD: Post-traumatic stress disorder
WTC: World Trade Center
WTCHR: World Trade Center Health Registry
